# Use of Artificial Intelligence to Detect Cardiac Rhythm Disturbances in Athletes: A Scoping Review

**DOI:** 10.1111/jvim.70257

**Published:** 2025-09-29

**Authors:** Amie Kapusniak, Natalia Medrano Lara, Peta L. Hitchens, Simon Bailey, Laura Nath, Samantha Franklin

**Affiliations:** ^1^ School of Animal and Veterinary Science University of Adelaide Roseworthy SA Australia; ^2^ Equine Centre, Melbourne Veterinary School Werribee VIC Australia

**Keywords:** arrhythmia, deep learning, electrocardiogram, machine learning, veterinary cardiology

## Abstract

**Background:**

Artificial intelligence (AI) is increasingly used to enhance electrocardiogram (ECG) interpretation in human medicine. In equine athletes, exercise‐associated arrhythmias are common and linked to sudden cardiac death at rates higher than in humans. However, ECG interpretation in horses remains time‐consuming and subjective, with the clinical relevance of mild rhythm disturbances often unclear.

**Objectives:**

Evaluate the application of AI to ECG interpretation for arrhythmia detection, with emphasis on current and potential use in athletic species, particularly horses.

**Animals:**

About 17 studies were included: 13 involving humans, 3 in horses, and 1 in dogs.

**Methods:**

A scoping review of relevant, peer‐reviewed studies published between 2000 and 2024 was conducted to identify research applying AI to ECG interpretation for arrhythmia detection. Studies were assessed for species, AI model type, diagnostic accuracy, and relevance to ECGs recorded during exercise. Primary outcomes included arrhythmia detection performance and applicability to veterinary medicine.

**Results:**

Deep learning models, including convolutional neural networks, achieved accuracies ranging from 79.4% to 98.6% in studies of humans. Research in horses showed encouraging results using restitution analysis and transfer learning approaches. However, small sample sizes and species‐specific ECG morphology remain major limitations to broader application in veterinary medicine.

**Conclusion and Clinical Importance:**

Artificial intelligence holds promise for enhancing the accuracy and efficiency of arrhythmia detection in ECGs of equine athletes. Development of species‐specific algorithms may facilitate real‐time monitoring of cardiac function during exercise, supporting improved cardiovascular assessment in athletic horses.

**Abbreviations**
AF: atrial fibrillation
AHS: athletic heart syndrome
AI: artificial intelligence
AIoT: artificial intelligence of things
ANN: artificial neural network
AUC: area under the curve
AUC‐ROC: area under the receiver operating characteristic curve
BiGRU: bidirectional gated recurrent unit
CNN: convolutional neural network
DL: deep learning
ECG: electrocardiogram
GRU: gated recurrent unit
HCM: hypertrophic cardiomyopathy
IoT: internet of things
k‐NN: k‐nearest neighbor
LSTM: long short‐term memory
ML: machine learning
MLP‐ANN: multilayer perceptron artificial neural network
NBETs: neural‐backed ensemble trees
NN: neural network
PAC: premature atrial contraction
pAF: paroxysmal atrial fibrillation
PVC: premature ventricular contraction
RBF: radial basis function
ReLU: rectified linear unit
RF: random forests
SPAR: symmetric projection attractor reconstruction
SVM: support vector machines

## 1 Introduction

Cardiac arrhythmias are of concern in athletic species because of their potential to negatively impact athletic performance and, on occasion, lead to collapse and sudden death. Atrial fibrillation (AF) is the most common clinically important arrhythmia in both humans and horses and has been reported to occur in 0.29%–4.9% of Thoroughbred and Standardbred racehorses [1, 2, 3]. The incidence of exercise‐associated sudden death in racing horses is estimated to be one to three per 10 000 starts [4], with over half of these fatalities attributed to suspected cardiac causes [5, 6]. This incidence has been estimated to be approximately 200 times higher than sudden deaths reported in human athletes [7, 8], highlighting the disproportionate risks faced by equine athletes. Necropsy examinations frequently fail to identify structural abnormalities, suggesting arrhythmogenic mechanisms as the most likely cause of sudden cardiac death (SCD) in horses [9, 10, 11, 12]. Early detection of horses that might be at increased risk of AF and SCD has been identified as an important area of research [4, 7].

Electrocardiography (ECG) remains the gold standard for arrhythmia detection, but its interpretation is time‐consuming, requires expertise [3], and suffers from poor intra‐ and interobserver reliability, especially during high‐speed exercise [13]. The clinical relevance of transient arrhythmias during exercise also remains unclear, adding further complexity to diagnosis and risk assessment.

Artificial intelligence (AI) has shown strong potential to automate ECG interpretation in human medicine, enhancing diagnostic accuracy and efficiency [14]. At the core of many AI systems are machine learning (ML) techniques such as convolutional neural networks (CNNs), which recognize complex, hierarchical patterns in ECG signals. These models can detect features ranging from basic waveform structures to subtle markers of arrhythmia, often outperforming traditional interpretation methods. Beyond classification, AI models also can predict arrhythmic risk and identify subclinical anomalies, offering continuous, real‐time cardiac monitoring [15, 16, 17, 18, 19, 20].

Despite these advances, the application of AI in equine cardiology remains limited. Horses have a distinct ventricular conduction system consisting of a deep Purkinje network that enables efficient electrical activation of their large hearts. As a result, their ECG morphology differs markedly from that of other species, most notably by exhibiting a dominant S wave instead of the prominent R wave typically observed in recordings from humans and dogs [21, 22, 23]. These species‐specific electrical conduction patterns present a challenge when attempting to apply ECG analysis software designed for humans and small animals to horses [24].

There is a critical need to develop species‐specific AI tools to interpret ECGs in equine athletes. Our scoping review aims to evaluate the current state of AI‐assisted ECG analysis for arrhythmia detection in athletic species, with a focus on horses. Our objectives were to map the existing literature, identify the types of AI models used, describe the arrhythmias studied, identify the methodological approaches taken, and summarize reported performance metrics. Given the emerging role of AI in both human and veterinary cardiology, our review provides a foundation to guide future research and support the development of AI‐assisted cardiac monitoring tools tailored to equine athletes.

## 2 Methods

### 2.1 Protocol and Registration

Our scoping review was conducted according to the Preferred Reporting Items for Systematic Reviews and Meta‐Analyses extension for Scoping Reviews (PRISMA‐ScR) guidelines (Data S1) [25].

### 2.2 Eligibility Criteria

Studies published between 2000 and 2024 that focused on the application of AI to ECG analysis in athletic species (humans, horses, and dogs) were included. The search strategy combined terms related to athletes and athletic animals, AI techniques, and ECG assessment. Non‐athletic individuals within these species were included in the study because cardiac pathology observed in non‐athletes also can be manifested in athletes and may be associated with athletic heart syndrome (AHS) [26, 27]. This inclusion allows for a more comprehensive analysis of cardiac conditions across the athletic spectrum. Studies on pediatric patients, differential diagnostic algorithms used by clinicians, non‐English language studies, and conference proceedings were excluded.

### 2.3 Information Sources and Search Strategy

The following databases were systematically searched for relevant studies: CAB Abstracts, Web of Science, Scopus, and PubMed. The search was last performed on September 2, 2024. The full search strategy was:

(athlet* OR “athlete's heart” OR horse OR equine OR racehorse OR thoroughbred OR endurance OR dog OR canine OR greyhound OR sled*) AND (“artificial intelligence” OR “machine learning” OR “neural network*” OR “transfer learning” OR “deep learning”) AND (electrocardiogram OR ECG OR heart OR cardiac).

Data was managed using Covidence [28]. After automatic removal of duplicates, two investigators (A.K. and N.M.L.) independently and blindly screened titles, abstracts, and full texts for eligibility. Disagreements were resolved by a third researcher (S.F.).

### 2.4 Data Charting Process and Data Items

A data charting form was developed and used to extract relevant information from included studies. Extracted data included general article information, study characteristics, participant details, AI exposure specifics, ECG and arrhythmia context, and key outcomes and findings. A detailed data extraction form is provided in Supporting Information S2 and Table S3.

### 2.5 Critical Appraisal of Individual Sources of Evidence

The quality of the included experimental studies was evaluated using a framework developed and customized to align with the objectives of our review. The assessment form is provided in Supporting Information S2 and Table S4. Nine domains were assessed: study design, participant selection, sample size, data collection, data analysis, outcome measures, reporting quality, ethical considerations, and generalizability. Each criterion was rated as “yes,” “no,” or “unsure” by the reviewers, with justifications provided for each decision. Studies were not excluded based on quality, but the appraisal was used to interpret findings in the context of methodological rigor and potential limitations.

### 2.6 Synthesis of Results

Studies were grouped by species and the types of AI used, and arrhythmias detected were summarized. For identified review articles, the included studies that potentially met the criteria were compared to the search results to identify any missed studies. In synthesizing the findings, the review aimed to identify and characterize existing AI algorithms and approaches used for ECG analysis in athletic species. It sought to evaluate the performance and limitations of AI applications in detecting various types of arrhythmias and assess the potential for transfer of learning from ECG datasets obtained from humans to ECG analysis of horses and dogs. Additionally, the review explored the challenges and opportunities in developing species‐specific AI algorithms for ECG interpretation. Finally, it evaluated the accuracy, efficiency, and expanded capabilities of AI‐driven ECG analysis compared to traditional methods.

## 3 Results

### 3.1 Selection of Sources of Evidence

**FIGURE 1:**
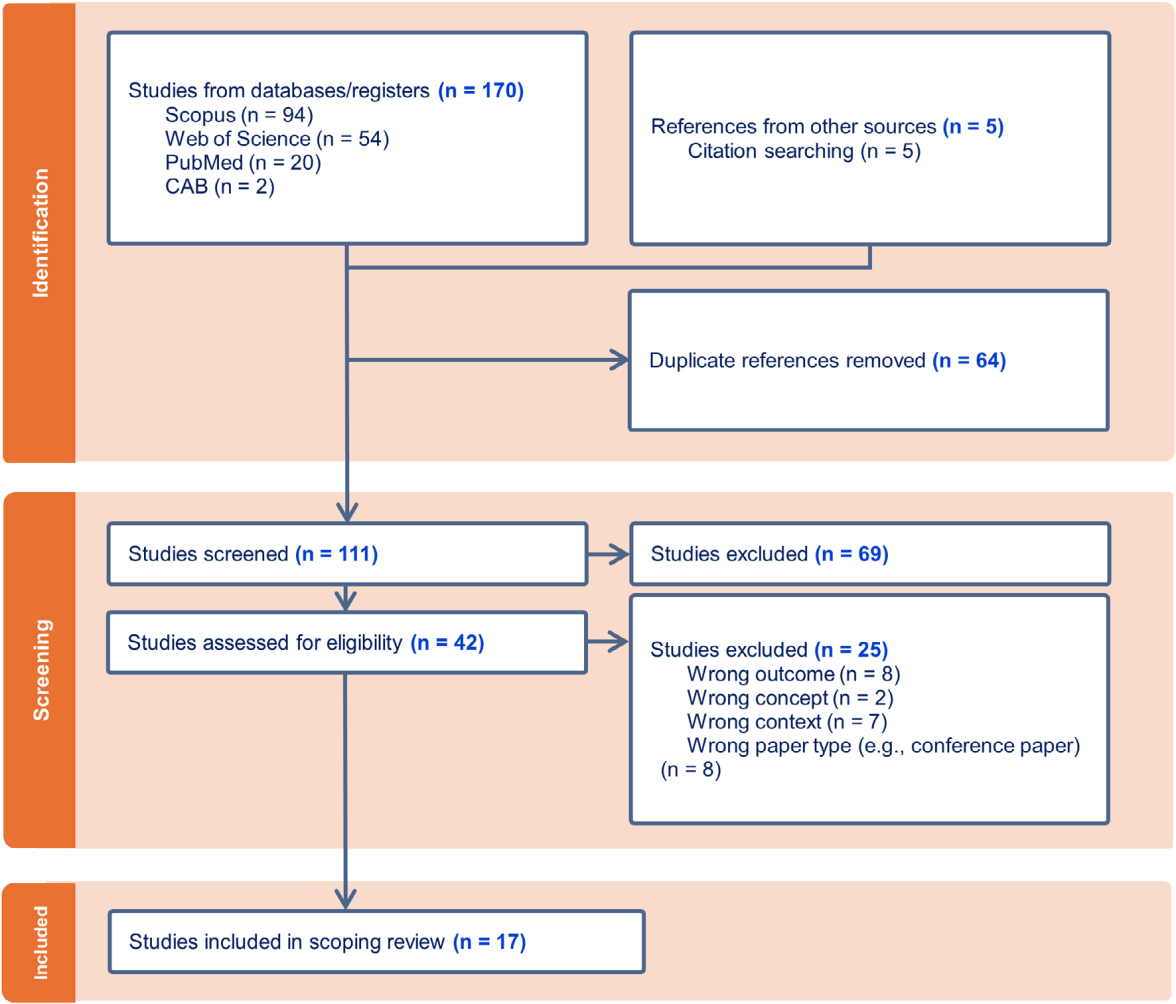
PRISMA flow diagram illustrating the scoping review process of studies using artificial intelligence to detect cardiac rhythm disturbances in athletic species from 2000 to 2024, including the number of studies identified, title and abstracts screened, full text articles assessed for eligibility and included in the final review, with reasons for exclusions at each stage.

In total, 174 peer‐reviewed journal articles from electronic databases and review article references were identified. After extraction, 17 studies met the eligibility criteria and were included in our review. Figure 1 presents a PRISMA flow diagram detailing the selection process.

### 3.2 Characteristics of Sources of Evidence

Of the 17 included studies, 13 focused on humans, consisting of 9 experimental studies (5 involving athletes; 4 involving non‐athletes) and 4 review articles (3 involving athletes; 1 involving non‐athletes). Eight studies specifically examined athletes (five experimental; three review articles). The four animal studies were all experimental, with three focusing on horses and one on dogs. None of the animal studies directly related to athletes or included ECG analysis during exercise. Overall, the studies included 13 experimental studies and four review articles. The earliest publication, a review, was published in 2014, whereas the first experimental study appeared in 2017. About 14 of the 16 studies were published after 2020.

### 3.3 Critical Appraisal Within Sources of Evidence

A critical appraisal was conducted for all included experimental studies and is summarized in Table 1. The appraisal assessed key quality indicators, including study design and participants, data quality and analysis, outcome reporting, ethics, and applicability. Table 1 also provides an overview of the species studied, ECG types used, and AI methods applied in each paper.

**TABLE 1:** Critical evaluation of experimental studies (*n* = 13) using AI for arrhythmia detection in humans, horses, and dogs (2000–2024).

Study				Critical appraisal								
				Study design and participants			Data quality and analysis		Outcomes and reporting		Ethics and applicability	
Study	Species	ECG type	AI method	Study design	Participant selection	Sample size	Data collection	Data analysis	Outcome measures	Reporting quality	Ethical approval	Generalizability
Adetiba et al. [29]	Human	Ambulatory	MLP‐ANN	Experimental non‐randomized, prospective	𝞆	✓	✓	✓	✓	✓	?	𝞆
Castillo‐Atoche et al. [16]	Human	Ambulatory	CNN	Experimental non‐randomized, prospective	𝞆	✓	✓	✓	✓	✓	✓	✓
Ji and Zhu [30]	Human	Resting	CNN + GRU	Observational non‐randomized, retrospective	?	✓	✓	✓	✓	✓	✓	✓
Munoz‐Macho et al. [31]	Human	Ambulatory	RF, SVM, CNN	Experimental non‐randomized, prospective	𝞆	✓	✓	✓	✓	✓	✓	✓
Zhuang et al. [17]	Human	Resting	CNN, LSTM	Observational non‐randomized, retrospective	𝞆	𝞆	𝞆	✓	✓	𝞆	𝞆	?
Attia et al. [18]	Human	Resting	CNN	Observational non‐randomized, retrospective	✓	✓	✓	✓	✓	✓	✓	?
Jo et al. [32]	Human	Resting	NBETs	Observational non‐randomized, retrospective	✓	✓	✓	✓	✓	✓	✓	✓
Kumar et al. [33]	Human	Ambulatory	Enhanced CNN	Observational non‐randomized, retrospective	𝞆	✓	✓	✓	✓	✓	✓	✓
Wang and Qin [34]	Human	Resting	CNN + BiGRU	Observational non‐randomized, retrospective	*?*	✓	✓	✓	✓	✓	✓	✓
Huang et al. [35]	Equine	Resting	k‐NN	Experimental non‐randomized, prospective	✓	✓	✓	✓	✓	✓	✓	✓
Huang et al. [36]	Equine	Resting	SVM, k‐NN	Experimental non‐randomized, prospective	✓	✓	✓	✓	✓	✓	✓	?
Van Steenkiste et al. [24]	Equine	Resting	CNN	Observational non‐randomized, retrospective	𝞆	✓	✓	✓	✓	✓	✓	✓
Flanders et al. [37]	Canine	Ambulatory	Neural network	Observational non‐randomized, retrospective	𝞆	✓	✓	✓	✓	✓	𝞆	✓

*Note:* ✓ indicates the criteria was met; 𝞆 indicates the criteria was not met; ? indicates that the authors were unsure if the criteria were met.

Abbreviations: BiGRU: bidirectional gated recurrent unit; GRU: gated recurrent unit; k‐NN: k‐nearest neighbor; LSTM: long short‐term memory; MLP‐ANN: multilayer perceptron artificial neural network; NBETs: neural‐backed ensemble trees; NN: neural network; RBF: radial basis function; ReLU: rectified linear unit; RF: random forests; SPAR: symmetric projection attractor reconstruction; SVM: support vector machines.

### 3.4 Results of Individual Sources of Evidence

The findings from each included source are summarized in Tables 2 and 3. Table 2 outlines the experimental studies, providing details on study objectives, cardiac conditions detected, key objectives, and relevant performance metrics such as sensitivity, specificity, and accuracy. Table 3 summarizes the review articles, presenting their overarching objectives, thematic focus, and key insights.

**TABLE 2:** A summary of objectives and outcomes of experimental studies (*n* = 13) from 2000 to 2024 examining the use of AI for detecting cardiac rhythm disturbances in athletic species, organized in alphabetical order by species.

Species	Study	Objective	Condition(s) detected	Findings	Performance metrics
**Humans** athletes	Adetiba et al. [29]	Develop an AI‐driven ECG system for automated heart defect detection.	Bradycardia Tachycardia HCM	ANN can be used for automated detection of heart defects in athletes based on ECG data.	Accuracy = 90%
	Castillo‐Atoche et al. [16]	Propose a wearable AIoT arrhythmia detection system for sports.	Not described	Detect arrhythmias in real time with low power use, enabling continuous ECG monitoring in athletic settings.	Accuracy = 98.6%
	Ji and Zhu [30]	Introduce CNN‐GRU model for ECG classification and exercise analysis.	Normal Supraventricular Ventricular Fusion beats	ECG classification achieved high accuracy in classifying ECG signals.	Accuracy = 99.47–100%
	Munoz‐Macho et al. [31]	Create an ECG database for football players and test ML for diagnosis.	Sinus bradycardia	Effectively detected sinus bradycardia with varied accuracy depending on model.	Accuracy = 95.33–100%
	Zhuang et al. [17]	Develop DL‐based arrhythmia diagnosis for young martial arts athletes.	Not specifically described	Improved arrhythmia detection accuracy and robustness by addressing confounders and temporal variations.	Accuracy = 87.01%–97.02%
**Humans** non‐athletes	Attia et al. [18]	Develop AI‐enabled ECG algorithm to detect AF.	AF	Detect AF during sinus rhythm, enabling early detection of at‐risk individuals.	Accuracy = 79.4% Sensitivity = 79% Specificity = 79.5%
	Jo et al. [32]	Develop and validate an explainable deep learning model for AF.	AF	Strong diagnostic performance for AF detection; Explainable AI improved interpretable outputs for clinical use.	Accuracy = 99.2%
	Kumar et al. [33]	Develop IoT‐based arrhythmia classification model.	Not specifically described	Effectively classified arrhythmias from IoT‐collected ECG data.	Accuracy = 95:33% Sensitivity = 94.92% Specificity = 97.57%
	Wang and Qin [34]	Design attention‐based BiGRU‐CNN for AF detection.	AF	Model enhances AF detection accuracy by combining convolutional and recurrent NN.	Accuracy = 97.7%–98.3%
Equine	Huang et al. [35]	Detect pAF using restitution analysis.	pAF	ECG restitution and ML can detect pAF with equine athletes, with relevance to human arrhythmia detection.	AUC ~0.8 for predicting pAF
	Huang et al. [36]	Detect pAF using SPAR & ML	pAF	SPAR with ML detected pAF from normal sinus rhythm.	Accuracy = 89%
	Van Steenkiste et al. [24]	Create beat‐to‐beat ECG analysis system using transfer learning.	Normal PVC PAC Noise	Parallel neural network enabled ECG transfer learning from humans to horses.	Accuracy = 97.1%
Canine	Flanders et al. [37]	Identify sinus node dysfunction using ML and Poincare plots.	Sinus node dysfunction	ML reported to distinguish sinus node dysfunction.	Not described.

Abbreviations: AIoT: artificial intelligence of things; AUC: area under the curve; HCM: hypertrophic cardiomyopathy; PAC: premature atrial contraction; pAF: paroxysmal atrial fibrillation; PVC: premature ventricular complex.

**TABLE 3:** A summary of objectives and outcomes of review articles from 2000 to 2024 examining the use of AI for detecting cardiac rhythm disturbances in athletes (A) and non‐athletic (N) humans.

Study	Focus	Objective	Findings
Bellfield et al. [38]	A	Review ML techniques used in athlete's heart research and their implementation.	ML shows potential to improve understanding, treatment and disease management in athlete's heart research.
Chang [39]	A	Discuss the controversy around ECG screening for SCD in young athletes and propose an AI solution.	Proposes using ANN to improve ECG interpretation for athlete screening.
Palermi et al. [40]	A	Explore AI applications in sports cardiology, including imaging, genetics and wearable devices.	Proposes using ANN to enhance ECG interpretation for athlete screening.
Attia et al. [14]	N	Review AI applications in ECG interpretation for cardiac screening and its impact on cardiovascular medicine.	AI transforms ECGs into tools for detecting cardiac and non‐cardiac diseases, including left ventricular dysfunction and AF.

### 3.5 Synthesis of Results

**FIGURE 2:**
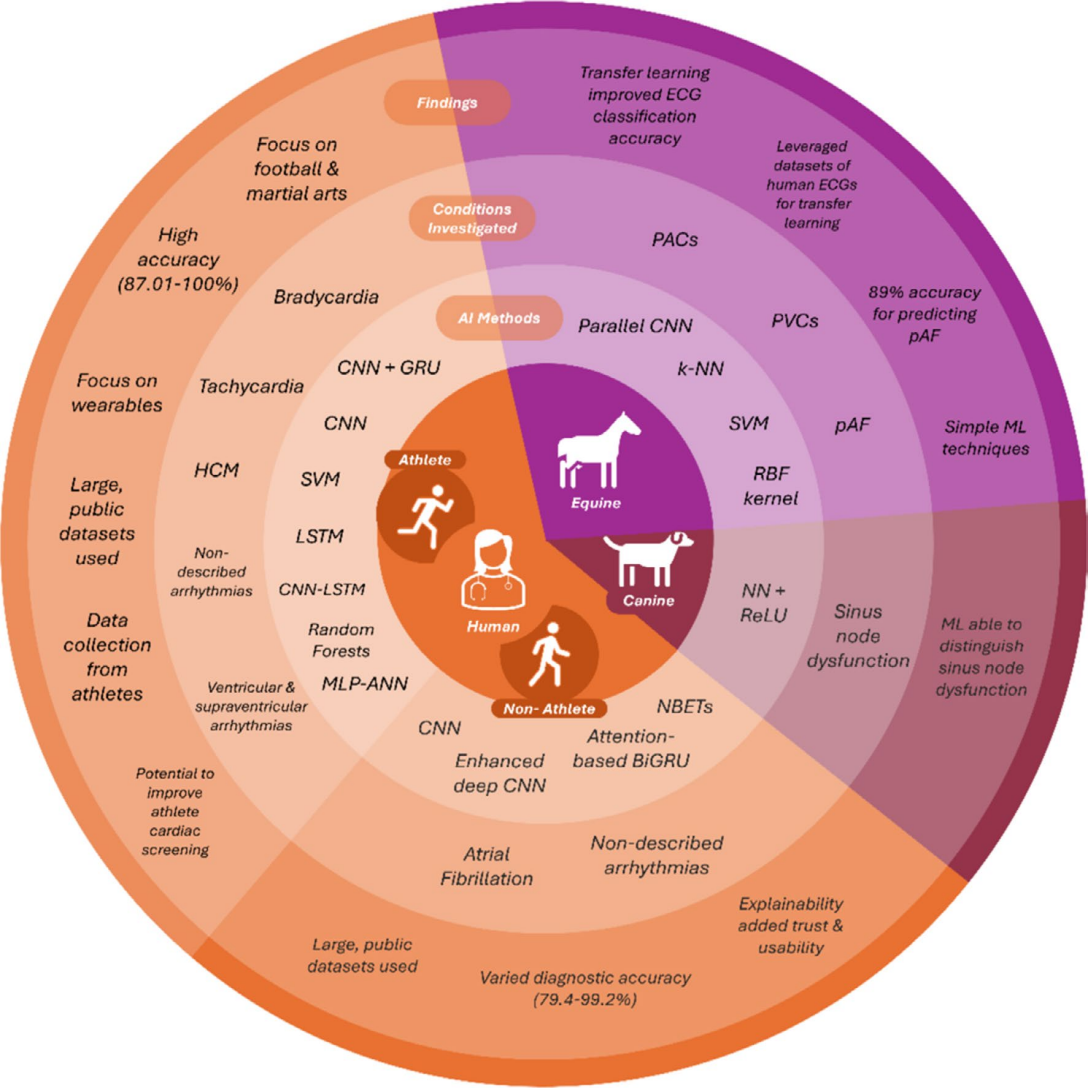
Synthesis of results across studies from 2000 to 2024 examining the use of AI detecting cardiac rhythm disturbances in human athletes and non‐athletes (orange), horses (purple) and dogs (maroon), summarizing key findings, trends and comparisons in AI applications for arrhythmia detection.

The synthesis of findings, presented in Figure 2, highlights the diverse applications of AI for ECG analysis and arrhythmia detection across species, with distinct trends and objectives depending on the target population.

For human non‐athletes, AI has primarily been employed to detect AF, leveraging advanced techniques such as CNNs and attention‐based bidirectional gated recurrent unit (BiGRU) models. These approaches had good diagnostic performance, with accuracy exceeding 90% in most studies (3/4) [32, 33, 34], and demonstrated potential for real‐time monitoring and improved clinical trust through explainable AI models [32]. For human athletes, the emphasis focused on classifying arrhythmias such as bradycardia [29, 31], tachycardia [29], and premature complexes [30]. Studies utilized neural networks (e.g., CNN, artificial neural networks [ANN], CNN with a gated recurrent unit [CNN‐GRU]) and ensemble ML models, achieving classification accuracies as high as 99.56% [30].

In horses, research utilized methods such as k‐nearest neighbor's (k‐NN) [35, 36], support vector machines (SVM) [36], and parallel CNNs, where transfer learning improved ECG classification accuracy from 92.6% to 97.1% [24]. However, challenges such as the unique morphology of ECGs in horses and small, species‐specific datasets limited generalizability. The study in dogs applied supervised ML with rectified linear unit (ReLU) activation functions, combined with Poincaré plots, to identify sinus node dysfunction [37].

Across all species, CNNs and ANNs emerged as dominant techniques, used in 9/13 experimental studies, achieving medium to high accuracy in arrhythmia detection (79.4%–98.6%). Wearable technologies were highlighted in multiple studies, particularly in athletes [16, 29, 40]. These technologies enabled continuous, real‐time data collection, which contributed to enhanced detection of arrhythmias. Convolutional neural networks and ANNs demonstrated adaptability across different species, with high performance in detecting arrhythmias from ECG data, particularly when large, diverse datasets were used. The ECGs across all species were recorded either at rest (7/13) or during ambulatory monitoring (6/13), but the level of exercise during ambulatory monitoring was not specified. None of the studies examined ECGs specifically recorded during exercise or evaluated the AI's performance in this context.

## 4 Discussion

Artificial intelligence is rapidly transforming the field of cardiology, offering innovative tools for detecting cardiac rhythm disturbances through ECG analysis. Although studies are few in number, much of the existing research focuses on applications in humans, leaving the potential for AI in veterinary cardiology, particularly in athletic species such as horses, largely underexplored. Our review examined the current state of AI applications in ECG analysis, mapping the methods, arrhythmias studied, and performance metrics reported in the literature.

Our findings highlight a growing interest in AI‐based ECG analysis, with the majority of studies (14/17) published since 2020, using human athletes or non‐athletes (13/17). Most of the evidence was derived from retrospective analyses of large ECG datasets (9/17), supplemented by reviews (4/17) and prospective studies (4/17). In the hierarchy of evidence‐based medicine, most of these study designs are considered lower levels of evidence. Although they offer valuable insight into AI applications in cardiology, they lack the methodological rigor of randomized controlled trials, which provide stronger evidence for clinical decision‐making. Commonly reported performance metrics included accuracy, sensitivity, specificity, and F1 score, but there was a lack of consistency in the reported findings across studies. The F1 score is a measure of a model's accuracy that balances sensitivity and precision, offering a single metric that accounts for both false positives and false negatives. In the experimental studies, AI models showed sensitivity ranging from 79.0% to 100%, specificity from 79.5% to 100%, accuracy from 79.4% to 100%, and F1 scores ranging from 39.2% to 100%, depending on the study. The studies in horses had promising results, with the k‐NN model achieving an area under the curve (AUC) of approximately 0.8 for predicting paroxysmal AF (pAF) [35]. When mathematical methods were combined with ML techniques, they achieved an accuracy of 89.0% and an AUC of 0.98 [36]. Additionally, transfer learning enhanced ECG classification accuracy from 92.6% (in the dataset from horses without transfer learning) to 97.1% (with transfer learning) [24]. Notably, most of the studies from horses (2/3) relied on simple ML techniques rather than more advanced deep learning (DL) approaches used in studies of humans. The study of dogs showed effective diagnosis using both supervised ML and Poincaré density grid methods but did not provide further performance metrics [37]. Direct comparison of the models is challenging because of differences in reporting, including variations in the reported performance metrics. The absence of standardized reporting guidelines for AI applications in ECG analysis may have contributed to these inconsistencies, making it difficult to objectively assess and compare the effectiveness of different models used across studies and species. Nonetheless, these findings suggest that for human athletes, AI‐based ECG analysis could enhance pre‐participation screening [39], health monitoring [14], and risk stratification for SCD [39]. In veterinary applications, AI shows potential to minimize inter‐observer variability and detect subtle arrhythmias, particularly during exercise in horses where traditional methods often fail [24].

Understanding whether AI models are intended for screening or diagnostic use is essential when interpreting their performance. Screening tools prioritize sensitivity to detect arrhythmias early, accepting more false positives, whereas diagnostic tools focus on specificity to avoid unnecessary intervention [41]. This trade‐off is critical in both human and equine athletes, where misclassification can have serious consequences. Most reviewed models reported higher specificity than sensitivity [17, 18, 29, 32, 33], likely reflecting a training bias toward diagnostic accuracy. This pattern likely reflects a training bias toward minimizing false positives, aligning more with diagnostic rather than screening goals. However, in the context of athletes, such as pre‐competition screening, higher sensitivity may be preferable to avoid missing life‐threatening arrhythmias. Future AI models should have their intended clinical role defined and their performance metrics tailored accordingly. Notably, few studies validated models in real‐world or exercise settings [16, 29], limiting their applicability to populations of athletes. Although no fixed thresholds exist, sensitivity and specificity > 80% generally are desirable for arrhythmia detection [41, 42, 43, 44], with the optimal balance depending on the clinical context and consequences of misclassification.

In the reviewed studies, AI models were commonly benchmarked against established methods, including public cardiac databases such as the MIT‐BIH arrhythmia database and expert clinician interpretation. However, variability in ECG quality, annotation standards, and arrhythmia types affected reported outcomes. Although these reference databases are well established in human medicine, equivalent standards are lacking in veterinary cardiology. These findings emphasize the need to tailor performance expectations and validation strategies to the model's clinical role. They also highlight the importance of standardized evaluation frameworks that distinguish between the goals of screening and diagnosis.

Convoluted neural networks and other DL models emerged as the predominant methods (used in 11/13 experimental studies), demonstrating high accuracy in detecting arrhythmias, such as AF [32, 34]. Convoluted neural networks function analogously to a skilled cardiologist, autonomously learning and identifying complex patterns in the ECG data using a hierarchical structure. The initial layers identify basic shapes and lines, like a cardiologist's initial glance; intermediate layers perform a more detailed analysis, recognizing specific waveform features; and the final layers integrate this information to deliver a diagnostic decision, much like the overall assessment of an expert [15]. This structure enables efficient, large‐scale ECG analysis, making CNNs well suited for both applications in human and veterinary medicine. However, biases can arise if training datasets lack diversity or disproportionately represent certain arrhythmias, potentially impairing performance in broader applications. Preprocessing methods, such as segmentation or filtering, also may influence feature detection, leading to false positives or missed diagnoses [15]. These limitations highlight the need for well‐curated datasets and robust validation to ensure reliability and generalizability.

A stark contrast in sample sizes between studies of humans and animals was observed. Studies of humans typically involved large datasets, with some utilizing over 100 000 ECG recordings [18, 32], whereas veterinary studies were limited to much smaller sample sizes. For instance, one study in horses analyzed only 26 400 heartbeats [24] and the study of dogs included 26 ECGs [37]. This disparity in sample sizes represents an important limitation in veterinary AI applications, potentially affecting the robustness and generalizability of the models developed for ECG analysis in animals.

The included studies indicated a limitation in current ML applications for ECG analysis in athletes. Although the reviewed experimental studies analyzed ECG recordings obtained at rest (7/13) or through ambulatory monitoring (6/13), none examined ECG data collected during exercise. Resting ECGs are valuable in clinical practice, offering a non‐invasive, accessible tool for identifying indicators of cardiac abnormalities without provoking arrhythmias. Studies found that ML applied to normal sinus rhythm could screen for pAF in horses [35, 36]. However, the utility of resting ECGs to predict exercising arrhythmias beyond pAF remains to be investigated. Many arrhythmias, particularly in athletes, are manifested during or immediately after physical exertion rather than at rest [45]. Exercise‐induced arrhythmias, such as ventricular ectopy and supraventricular tachycardias, often emerge in response to changes in autonomic tone, heart rate variability, and cardiac workload during or immediately after physical exertion. These physiological changes can unmask underlying abnormalities or trigger arrhythmias not apparent at rest [27, 46]. Without exercise‐specific ECG data, it is difficult to evaluate how effectively ML models can detect these dynamic and often transient events. This limitation impacts both sensitivity and specificity because models trained solely on resting ECGs may fail to identify exertion‐related arrhythmias (low sensitivity) or incorrectly flag normal findings as abnormal (low specificity). This challenge is particularly pronounced in horses, where high heart rates during exercise can result in the merging of T and P waves, along with increased motion artifact, further complicating accurate arrhythmia detection [47]. These findings emphasize the need for future research to incorporate exercising ECGs and improve the clinical relevance and robustness of AI models in equine and human sports cardiology. The application of AI in ECG analysis of horses represents a promising but underexplored area, with only three (of 17) papers focusing on horses. Despite limited research, early findings show potential, with AI‐based models successfully classifying ECG complexes in horses [24] and predicting pAF based on recordings made during sinus rhythm [35]. These advancements are particularly encouraging given the unique challenges in ECG interpretation of horses, such as the prominent S peak and the tendency for P waves to overlap with T waves during exercise, which necessitate tailored AI models [24]. Limited annotated data further constrains veterinary models, but transfer learning appears to offer a practical solution. By leveraging pre‐trained models from ECG data of humans and fine‐tuning them for features specific to horses, transfer learning enhances AI performance in identifying complex patterns and addressing data scarcity. Expanding research into exercising horses, particularly at high intensities, is crucial for improving diagnostic precision and enabling real‐time assessments during athletic performance.

Our review had several limitations. The small number of studies on ECG analysis of horses and dogs limits the generalizability of findings to these species. The heterogeneity of AI techniques, datasets, and performance metrics reported complicates direct comparisons among studies. The lack of standardized reporting guidelines for AI applications in ECG analysis introduces further inconsistencies, and the risk of publication bias favoring positive results cannot be excluded. Additionally, some of the studies had methodological flaws (Table 2), including unjustified participant selection, lack of ethical consideration, and limited reporting of key performance metrics. These methodological shortcomings not only hinder reproducibility but also limit the reliability of the reported outcomes, emphasizing the need for more rigorous study design and comprehensive reporting standards in future research.

Future research should focus on developing species‐specific AI models tailored to ECG analysis of horses and dogs. Research on exercise ECG interpretation is particularly critical for horses, where current methods often fail to provide consistent results. Further exploring the potential for transfer learning from ECG datasets of humans could accelerate progress in veterinary applications [24]. Additionally, longitudinal studies are needed to assess the predictive capabilities of AI models for future cardiac events. Efforts to develop standardized reporting guidelines for AI studies in veterinary ECG analysis would enhance comparability and support more robust meta‐analyses. Integrating AI‐based ECG analysis with other diagnostic modalities, such as echocardiography, has the potential to enable more comprehensive cardiac assessments [38]. Additionally, the incorporation of AI‐enhanced ECG systems into wearable technologies could facilitate improved real‐time monitoring, particularly during exercise [16, 29]. Because these technologies continue to evolve, rigorous real‐world validation of AI models in clinical settings, along with careful consideration of ethical and regulatory frameworks, will be essential to ensure effective and responsible implementation [14].

In conclusion, AI has shown substantial potential to advance ECG analysis for arrhythmia detection, with CNNs and DL models generally demonstrating high accuracy and efficiency across human and veterinary applications. Despite promising results in human athletes, veterinary studies remain limited, particularly for horses where unique cardiac features pose challenges. Key priorities include developing species‐specific AI models, leveraging transfer learning, and investigating AI interpretation of ECG tracings acquired during exercise. Standardized reporting guidelines, larger datasets, and real‐world validation are crucial for future progress. Integrating AI with other diagnostic tools and wearable technologies offers transformative potential for both human and veterinary sports cardiology.

## Disclosure

Authors declare no off‐label use of antimicrobials.

## Ethics Statement

Authors declare no institutional animal care and use committee or other approval was needed. Authors declare human ethics approval was not needed.

## Conflicts of Interest

The authors declare no conflicts of interest.

## Supporting information


**Data S1:** jvim70257‐sup‐0001‐supinfo.docx.

**Data S2:** jvim70257‐sup‐0002‐supinfo.docx.
