# Supplementary material for: Use of Artificial Intelligence to Detect Cardiac Rhythm Disturbances in Athletes: A Scoping Review
Source: J Vet Intern Med. 2025 Sep 29;39(6):e70257. doi: 10.1111/jvim.70257 (PMC12477403; doi:10.1111/jvim.70257)
Supplement: Supplementary file 2 — Data S2: jvim70257‐sup‐0002‐supinfo.docx. [file JVIM-39-e70257-s002.docx]

**Supplementary Material 2**: Protocol for Scoping Review

*TITLE*: Using artificial intelligence to detect cardiac rhythm disturbances in athletic species: a scoping review and its implications for horses

**INTRODUCTION**

*RATIONALE:* Cardiac rhythm disturbances in athletic species, especially in horses, pose a risk for death. Although ECGs are essential for detecting arrhythmias, their interpretation often lacks consistency, making artificial intelligence a promising solution to improve accuracy and timely detection.

*OBJECTIVES:*

- Identify and characterise existing AI algorithms and approaches used for ECG analysis in athletic species.
- Evaluate the performance and limitations of AI applications in detecting various types of arrhythmias.
- Assess the potential for transfer learning from human ECG datasets to equine and canine ECG analysis.
- Explore the challenges and opportunities in developing species-specific AI algorithms for ECG interpretation.
- Evaluate the accuracy, efficiency, and expanded capabilities of AI-driven ECG analysis.

*RESEARCH QUESTION: “Can artificial intelligence be used to evaluate features of electrocardiograms (ECG) in athletic species to predict cardiac rhythm disturbances?”*

**METHODS**

*PROTCOL & REGISTRATION:* This scoping review will be conducted according to the Preferred Reporting Items for Systematic Reviews and Meta-Analyses extension for Scoping Reviews (PRISMA-ScR) guidelines[1].

*ELIGIBILITY CRITERIA:* The eligibility criteria are described in Table 1.

Table 1: Inclusion and exclusion criteria for articles to be review for the scoping review.

|  | *Included* | *Excluded* |
| --- | --- | --- |
| *Patients* | - Human patients - Human athletes - Horses - Dogs | - Paediatric patients |
| *Exposure* | - Artificial intelligence, machine intelligence, machine learning, computational intelligence, predictive technology, transfer learning, deep learning - Electrocardiogram, ECG | - Differential diagnostic algorithm used by clinicians |
| *Context* | - Cardiac rhythm disturbances - Arrythmias - Conduction disorders - Premature complexes | - Related to structural cardiac disease without cardiac rhythm disturbances |
| *Outcome* | - Using AI for ECG analysis | - Using AI for investigation of other cardiac diagnostics (e.g., echocardiography) |
| *Study Characteristics* | - Cohort, case-control or cross-sectional studies - Case series, case reports - Randomised controlled trials - Narrative reviews, meta-analyses | - Conference proceedings |
| *Language* | - English language studies | - Non-english language studies |
| *Publication Type* | - Peer reviewed publications - Studies published post-xc2000 | - Studies published pre-2000 |

*INFORMATION SOURCES & SEARCH:*

String Search: *(athlet* OR "athlete's heart" OR horse OR equine OR racehorse OR thoroughbred OR endurance OR dog OR canine OR greyhound OR sled*) AND ("artificial intelligence" OR "machine learning" OR "neural network*" OR "transfer learning" OR "deep learning") AND (electrocardiogram OR ECG OR heart OR cardiac)*

Table 2: Complete string searches for each of the databases to be utilised in the scoping review.

| Database | Hits | Complete Search String |
| --- | --- | --- |
| CAB Abstracts | 2 | ((TS=(athlete* OR "athletes heart" OR horse* OR equine OR racehorse* OR “race horse*” OR thoroughbred OR standardbred OR endurance OR dog OR canine OR greyhound OR sled-dog)) AND TS=("artificial intelligence" OR "machine learning" OR "neural network*" OR "transfer learning" OR "deep learning") AND TS=(electrocardio* OR ECG OR EKG))  Filters applied: English, from 2000-2024 |
| Web of Science | 54 | (((ALL=(athlete* OR “athletes heart: OR horse* OR equine OR racehorse* OR “race horse*” OR thoroughbred OR standardbred OR endurance OR dog OR canine OR greyhound OR sled-dog (topic)) AND ALL=(artificial intelligence OR machine learning OR neural network* OR transfer learning OR deep learning (topic)) AND ALL=(electrocardio* OR ECG OR EKG (topic))) and 1999 (Exclude – Publication Years) and English (Languages) |
| Scopus | 94 | ( TITLE-ABS-KEY ( athlete* OR "athletes heart" OR horse* OR equine OR racehorse* OR thoroughbred OR standardbred OR endurance OR dog OR canine OR greyhound OR sled-dog ) AND PUBYEAR > 1999 ) AND ( TITLE-ABS-KEY ( "artificial intelligence" OR "machine learning" OR "neural network*" OR "transfer learning" OR "deep learning" ) AND PUBYEAR > 1999 ) AND ( TITLE-ABS-KEY ( electrocardio* OR ECG OR EKG ) AND PUBYEAR > 1999 ) AND ( LIMIT-TO ( LANGUAGE , "English" ) ) |
| PubMed | 20 | (“athletes”[mh] OR athlete*[tiab] OR athletes heart[tiab] OR horse*[tiab] OR equine[tiab] OR racehorse*[tiab] OR race horses[tiab] OR thoroughbred[tiab] OR standardbred[tiab] OR endurance[tiab] OR dog[tiab] OR canine*[tiab] OR greyhound[tiab] OR sled-dog[tiab]) AND (“artificial intelligence”[mh] OR artificial intelligence[tiab] OR machine learning[tiab] OR neural network[tiab] OR transfer learning[tiab] OR deep learning[tiab]) AND (“electrocardiography”[mh] OR electrocardio*[tiab] OR ECG[tiab] OR EKG[tiab])  Filters: English, from 2000 – 2024 Sort by: Publication Date |

*SELECTION OF SOURCES OF EVIDENCE:* A comprehensive literature search will be conducted across the four databases using the predefined search terms. Search results will be imported into the Covidence platform, where duplicates will be automatically removed and manually verified by the investigators. Two investigators will independently and blindly screen titles, abstracts and full texts (as required) to determine eligibility. Any disagreements will be resolved through consultation with a third investigator.

*DATA CHARTING PROCESS & DATA ITEM:* Upon reviewing the full-text publications, the study characteristics and relevant information will be systematically documented in the table presented in Table 3. Data will be extracted for the following variables: study characteristics, population details, AI methods, performance metrics and clinical context. Assumptions will be made where specific details are missing or unclear, such as categorising non-specified deep learning models as convolutional neural networks (CNNs) or grouping arrhythmias under general categories when specific types are not reported. These data items will be extracted systematically to ensure consistency and transparency, facilitating a comprehensive synthesis of the use of AI in ECG based arrhythmia detection.

Table 3: Data extraction chart to be utilised for each eligible study in the scoping review.

| **Study ID** | **Study Name** | *Study 1* | *Study 2* | *Study 3, etc.* |
| --- | --- | --- | --- | --- |
| **Study Characteristics** | Main Objective: |  |  |  |
|  | Study Design: |  |  |  |
|  | Conflicts of Interest: |  |  |  |
| **Patients** | Sample Size: |  |  |  |
|  | Population: |  |  |  |
|  | Recruitment Method: |  |  |  |
| **Exposure**  AI Details | AI Algorithm: |  |  |  |
|  | Input Data: |  |  |  |
|  | Dataset Size/Source: |  |  |  |
|  | Performance Metrics:   - Accuracy: - Sensitivity: - Specificity: - Other Metrics: |  |  |  |
|  | Explainability Features: |  |  |  |
| **Context**  ECG & Arrhythmia Details | Arrhythmias Detected: |  |  |  |
|  | ECG Method |  |  |  |
|  | Data Setting: |  |  |  |
| **Outcomes & Findings** | Findings: |  |  |  |
|  | Clinical Utility: |  |  |  |
|  | Limitations: |  |  |  |
|  | Practice Implications: |  |  |  |
| **Quality Assessments & Standards** | Reporting Standards: |  |  |  |
|  | Ethical Considerations: |  |  |  |
| **Other Key Details** | Funding: |  |  |  |
|  | Future Directions: |  |  |  |

*CRITICAL APPRAISAL OF EVIDENCE:* A critical appraisal of the included experimental studies will be conducted to assess their quality and ensure findings are interpreted within the context of methodological rigor. An evaluation framework, customised to align with this review's objectives, was developed. The critical appraisal form (Table 4) evaluates key domains including study design, participant selection, data collection, outcome measures, sample size, data analysis, confounding factors, result reporting, ethical considerations, and generalisability. Each question will be answered as "yes," "no," or "unsure," with supporting comments provided for each decision.

Table 4: Critical evaluation criteria for each eligible experimental study in the scoping review.

| **Category** | *Study 1* | *Study 2* | *Study 3* | *Study 4, etc.* |
| --- | --- | --- | --- | --- |
| **Study Design:** Was the study design appropriate for the research question? |  |  |  |  |
| **Participant Selection:** Were the inclusion and exclusion criteria clearly defined? Was the selection process appropriate to minimize bias? |  |  |  |  |
| **Data Collection:** Were the methods for data collection clearly described and standardized across all participants? |  |  |  |  |
| **Outcome Measures:** Were the outcome measures clearly defined and appropriate for the study objectives? |  |  |  |  |
| **Sample Size:** Was the sample size adequate and justified? |  |  |  |  |
| **Data Analysis:** Were appropriate statistical methods used for the analysis? |  |  |  |  |
| **Confounding Factors:** Were potential confounding factors identified and addressed in the analysis? |  |  |  |  |
| **Reporting of Results:** Were the results reported comprehensively and clearly? |  |  |  |  |
| **Ethical Considerations:** Were ethical approvals obtained and informed consent procedures followed? |  |  |  |  |
| **Generalisability:** Are the findings generalisable to the target population? |  |  |  |  |

*REFERENCES:*

[1] Tricco, A.C., Lillie, E., Zarin, W., O'Brien, K.K., Colquhoun, H., Levac, D., Moher, D., Peters, M.D., Horsley, T. and Weeks, L. (2018) PRISMA extension for scoping reviews (PRISMA-ScR): checklist and explanation. *Annals of internal medicine* **169**, 467-473.
